# Using support vector machines to improve elemental ion identification in macromolecular crystal structures

**DOI:** 10.1107/S1399004715004241

**Published:** 2015-04-25

**Authors:** Nader Morshed, Nathaniel Echols, Paul D. Adams

**Affiliations:** a College of Letters and Science, University of California, Berkeley, CA 94720, USA; b Physical Biosciences Division, Lawrence Berkeley National Laboratory, Berkeley, CA 94720, USA; c Department of Bioengineering, University of California, Berkeley, CA 94720, USA

**Keywords:** elemental ion identification, support vector machines, model building

## Abstract

In the process of macromolecular model building, crystallographers must examine electron density for isolated atoms and differentiate sites containing structured solvent molecules from those containing elemental ions. This task requires specific knowledge of metal-binding chemistry and scattering properties and is prone to error. A method has previously been described to identify ions based on manually chosen criteria for a number of elements. Here, the use of support vector machines (SVMs) to automatically classify isolated atoms as either solvent or one of various ions is described. Two data sets of protein crystal structures, one containing manually curated structures deposited with anomalous diffraction data and another with automatically filtered, high-resolution structures, were constructed. On the manually curated data set, an SVM classifier was able to distinguish calcium from manganese, zinc, iron and nickel, as well as all five of these ions from water molecules, with a high degree of accuracy. Additionally, SVMs trained on the automatically curated set of high-resolution structures were able to successfully classify most common elemental ions in an independent validation test set. This method is readily extensible to other elemental ions and can also be used in conjunction with previous methods based on *a priori* expectations of the chemical environment and X-ray scattering.

## Abstract 2

A method to automatically identify possible elemental ions in X-ray crystal structures has been extended to use support vector machine (SVM) classifiers trained on selected structures in the PDB, with significantly improved sensitivity over manually encoded heuristics.

## 1. Introduction   

Elemental ions are essential for the function of many proteins and are also present at high concentrations in many crystallization solutions. The identification of these ions in crystal structures requires the careful analysis of both the chemical environment and scattering properties, usually in comparison to water molecules. Restrictions on the chemical environment of an ion can include coordination by certain elements and at distances that are expected to balance the charge of the ion (Nayal & Di Cera, 1996 ▶; Müller *et al.*, 2003 ▶; Dokmanić *et al.*, 2008 ▶; Zheng *et al.*, 2008 ▶; Brown, 2009 ▶; Harding *et al.*, 2010 ▶; Carugo, 2014 ▶). Abnormal *B* factors (atomic displacement parameters) or peaks in the *mF*_o_ − *DF*_c_ map can be used to coarsely filter candidates by number of electrons. When metals bind in similar chemical environments, one can also use anomalous scattering information to narrow down the list of candidate ions (Ascone & Strange, 2009 ▶; Harding *et al.*, 2010 ▶; Echols *et al.*, 2014 ▶). Crystallographers usually manually apply these selection criteria, starting with the sites flagged by a solvent-picking procedure. Ions are labeled based on the researcher’s intuition, some subset of the above listed features and other previously known biochemical information.

A complete solution for automated structure determination should include correctly building ions into the structure, but this task is made difficult by the fact that many metals bind the same structural features of a protein, can adopt similar co­ordination geometries and have similar scattering properties (Zheng *et al.*, 2008 ▶; Harding *et al.*, 2010 ▶). These ambiguities can lead to errors in both the automated and the manual interpretation of diffraction data. Indeed, multiple groups have found evidence for a substantial number of structures with suspicious metal assignments (Chruszcz *et al.*, 2010 ▶; Dauter *et al.*, 2014 ▶; Echols *et al.*, 2014 ▶; Zheng *et al.*, 2014 ▶).

Previously, we have summarized the useful criteria in ion identification and have described a method for automatically identifying ions as part of the *PHENIX* software for automated macromolecular crystallography (Adams *et al.*, 2010 ▶; Echols *et al.*, 2014 ▶). This method identifies ions using a tree-like set of manually determined rules, henceforth described as a decision tree. These rules consist of a number of constraints on features in the chemical environments and scattering properties. The parameters for these constraints were taken from reported values in the literature and then optimized on structures deposited by the Joint Center for Structural Genomics (JCSG; Elsliger *et al.*, 2010 ▶). However, the design of this procedure limits its sensitivity and requires developers to manually determine the parameters for each new element. Consequently, it has only received thorough optimization and validation on zinc and calcium. Furthermore, rather than weighing all features of a site to find a unified probabilistic score, the method merely returns a list of compatible ions whose constraints have not been violated.

**Figure 1:**
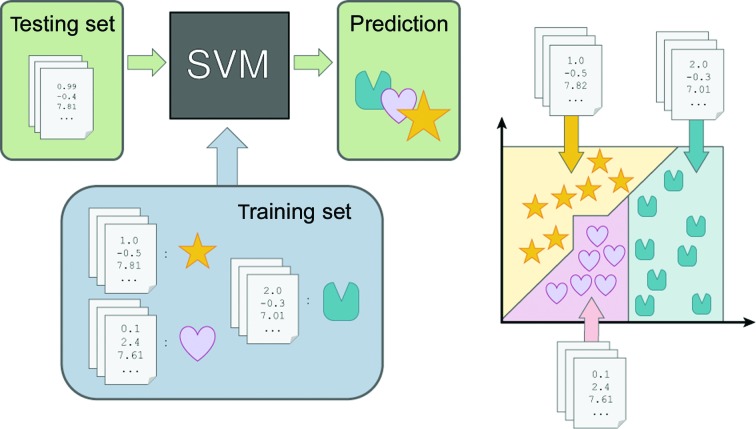
An illustration of the general design of support vector machines (SVMs). SVMs are initially trained by input of examples of each class and their associated feature values. A trained SVM is then able to predict the identity of future objects based on their feature values (left). The underlying mechanism of classification involves finding the set of hyperplanes that best divide the space between examples in *N* dimensions, where *N* is the number of values in a feature. Here, this is depicted as lines dividing two-dimensional space (right). Other types of SVMs allow nonlinear functions to divide this space.

In order to address these issues, we have investigated using machine-learning algorithms to automatically detect the patterns that differentiate ions from one another. In the context of structural biology, these methods have shown success in the analysis of crystallization images (Pan *et al.*, 2006 ▶) as well as in the prediction of binding and functional sites from both sequence (Lippi *et al.*, 2012 ▶; Carugo, 2008 ▶) and structure (Brylinski & Skolnick, 2011 ▶; Buturovic *et al.*, 2014 ▶), structural polymorphism (Takaya *et al.*, 2013 ▶), the results of mutation experiments (Wei *et al.*, 2013 ▶) and model building into electron density (Holton *et al.*, 2000 ▶; Gopal *et al.*, 2007 ▶). Here, we present an advance upon our previous method, in which we use support vector machines (SVMs) to classify sites as either water or one of various elemental ions. SVMs are a class of machine-learning algorithms that take the approach of treating input vectors as points in *N*-dimensional space, where *N* is the number of features used to describe the inputs. The best set of hyperplanes that divide the space between these points is determined and the confidence of the prediction is derived from the distance of a point to the nearest hyperplane (Wu *et al.*, 2007 ▶; Fig. 1 ▶).

We have compiled several large sets of ion-containing structures from the Protein Data Bank (PDB; Berman *et al.*, 2000 ▶) and trained SVMs on their ion and water sites using features from both the chemical and scattering environments. Three independent types of data sets were used: (i) manually curated structures across a broad resolution range, (ii) automatically curated high-resolution structures and (iii) a more stringent version of (ii) in which individual ion sites were subjected to tighter thresholds on allowed binding and scattering features. The SVMs exhibit very high sensitivity for all of the ‘heavy’ (fourth-period) ions (calcium and transition metals) with very few mislabeled water molecules. They were usually able to reliably distinguish different classes of elements, but as expected have difficulty in distinguishing similar transition metals. In high-resolution structures they are able to detect all common ions except sodium, with up to twice the sensitivity of the previous method. When combined with a simple filter for quality control in the SVM predictions, we found that the true-positive rate almost doubled, while the false-positive rate was no higher than when using a decision tree. On manual inspection, we also found that these SVMs uncovered many un­modeled ions. These findings highlight the value of automated methods in correctly labeling ions in future structures before they are deposited in the PDB. Our methods are easily extensible to support additional, rarer elements as more structures become available.

## 2. Methods   

### 2.1. Data sets   

To train and test the SVMs, we assembled three separate collections of structures: (i) a curated structure set including metal-bound protein structures that were deposited with anomalous diffraction data and manually inspected to ensure that ions were not mislabeled, (ii) an automatically curated structure set containing all ion-bound sequence-unique protein structures that met a certain resolution limit and (iii) a more stringently curated structure set in which the ion sites from the training set of (ii) were passed through a simple set of input filters. Within each of these data sets, the structures were randomly assigned to independent training and test sets. To train each SVM, we performed feature selection and scanned SVM parameters using cross-validation within the training set. We tested the best-performing SVM and set of parameters on the test set, which was kept separate during the process of training.

#### 2.1.1. Curated structure set   

To build our curated training and test sets, we first collected a list of X-ray crystal structures in the PDB that (i) contained at least one metal of interest, (ii) contained diffraction data with separate anomalous pairs and (iii) had a resolution of or better than 3 Å. For this analysis we restricted the choice of ions to the most common heavier elements (Ca, Mn, Fe, Zn and Ni). We then re-refined the structures using *phenix.refine* (Afonine *et al.*, 2012 ▶) and manually examined them using *Coot* (Emsley *et al.*, 2010 ▶). We retained the structures where the metal assignments appeared to be reasonable based on any noted crystallization conditions or other experimental information and previously described patterns in metal binding and anomalous scattering (Zheng *et al.*, 2008 ▶, 2014 ▶; Harding *et al.*, 2010 ▶; Echols *et al.*, 2014 ▶); however, we did not screen any sites based on their coordination geometry. Structures were assigned to the training set until at least 50 examples of each site were present, after which we attempted to evenly represent each metal in the training and test sets.

#### 2.1.2. Automatically collected and curated high-resolution structure set   

We constructed separate high-resolution structure sets for sodium, magnesium, chloride, potassium, calcium, manganese, iron, cobalt, nickel, copper, zinc and cadmium ions. For each set, we collected a list of X-ray crystal structures from the PDB that (i) contained at least one site with that element at any charge, (ii) had a resolution of 2.0 Å or better (1.5 Å for Na^+^, Mg^2+^, Cl^−^, Ca^2+^ and Zn^2+^), (iii) included deposited diffraction data and (iv) included only protein in their macromolecular content. In order to obtain as large a sample size as possible, we did not require that anomalous data be available. Redundant structures were filtered out using a 90% sequence-identity cutoff. For SVM benchmarking, we randomly assigned 200 chloride-containing structures to the test set. This number was 100 for sodium, magnesium, potassium, calcium, manganese, iron and zinc, and 50 for cobalt, nickel, copper and cadmium. These numbers gave a split of around 2:1 between the training and test set for all ions. All remaining structures were assigned to the training set. Note that the structures in this data set were not filtered for having anomalous data present, and thus no anomalous scattering information was included when training the SVM on this data set.

#### 2.1.3. Stringently curated high-resolution structure set   

To test the effect of simple, automated quality-control filters on the training inputs, we also extracted a third training set from the high-resolution structures. The training ion sites in this set were excluded if they failed to pass a few simple filters: (i) sodium ions were required to have bond-valence sums (see below) between 0.6 and 1.4, (ii) magnesium ions were required to have bond-valence sums between 1.5 and 2.5, (iii) all ions were required to have a σ level in the *mF*_o_ map (generated directly from **F**_obs_ structure factors) greater than or equal to 1 and (iv) all ions were required to have an *mF*_o_ − *DF*_c_ peak height of at least 0, as measured below. No filters were applied to the test sites.

### 2.2. Structure processing   

For each structure in both the test and training sets, we replaced the ions in each structure with water molecules and refined the result using *phenix.refine* with the arguments waters=False refine.sites.individual=‘not water’. These options disabled the positional refinement of waters as well as the automatic deletion or placement of new waters. This kept all sites intact and prevent waters from being refined into heavier atom sites that had been anonymized. We discarded any structures where (i) the *R*_free_ increased by more than 0.04, (ii) the average bond-length deviation increased by more than 0.05 Å or (iii) the average bond-angle deviation increased by more than 0.57°. We then built a list of the scattering and chemical environments for each ion and water site in the refined structures. Alternate conformations of water molecules and ions were excluded from this list.

### 2.3. Classifier training   

#### 2.3.1. SVM setup   

To train each SVM, we first extracted quantitative features from each pair of scattering and chemical environment objects, as listed below. In order to prevent bias towards features with inherently larger ranges, we normalized their values to fit in the range −1 to 1, mapping the minimum and maximum values from each feature in the training set to −1 and 1, respectively. We then used *scikit-learn* v.0.13.1 (Pedregosa *et al.*, 2012 ▶) to perform recursive feature elimination with fivefold cross-validation (RFECV; Guyon *et al.*, 2002 ▶) for feature selection.

To generate the classifier, we used *LIBSVM* v.3.18 (Chang & Lin, 2011 ▶) to score SVMs trained with a range of values for the soft margin constant *C*. This parameter controls to what extent a hyperplane is affected by values at its margins, and it is important to keep it low to avoid overfitting (Ben-Hur & Weston, 2010 ▶). *C* values were tested at increasing orders of magnitude between 0.001 and 10 000. We discarded the sets of parameters that caused their SVM to take more than a week to be trained. Class weights were also calculated to give equal representation to each class of ions and prevent overtraining on water sites. We enabled shrinking heuristics, an option within *LIBSVM* that improves the speed of training on some data sets. For all sets of parameters, we trained the SVM using a linear kernel. As RFECV requires a fully trained SVM during its calculations, feature selection was re-calculated for each set of parameters. Each set of parameters were ranked according to the performance of the resulting SVM on the portion of the training set assigned to the cross-validation group. The optimal parameters were then used to train an SVM on the entire training set with the added option to calculate probability estimates.

This same procedure was run on each high-resolution data set using separate SVMs trained only to differentiate these ions from water molecules in the same structures. Owing to constraints on computation time, we limited the total number of water molecules in any one training set to a randomly selected subset of 150 000 sites. In the case of sodium as well as the merge of all high-resolution structures, this number was decreased to 100 000.

#### 2.3.2. Site features   

The features measured at a site can be divided into three groups: (i) X-ray scattering, (ii) the chemical environment and, optionally, (iii) anomalous scattering information. From the X-ray scattering environment, we included (i) the highest resolution of the diffraction data (*d*_min_) rounded to the nearest 0.5 Å, (ii) the height and spread of a Gaussian function fitted to the *mF*_o_ map, using points in a 1.6 Å radius around the site, (iii) the peak height in the *mF*_o_ − *DF*_c_ map measured *via* eight-point interpolation, (iv) the *B* factor of the atom divided by the average *B* factors of all structured water molecules in the model and (v) the occupancy of the atom.

From the chemical environment, we included (i) the presence of each geometric shape at the coordination site (see below), (ii) the number of coordinating atoms for each common coordination group and (iii) the bond-valence sum (BVS) and vector sum of bond valences (VECSUM) (Müller *et al.*, 2003 ▶), as calculated for each supported ion.

The list of coordination groups included carboxyl, amide, backbone N atoms and carbonyls, sulfate, phosphate, sulfide, disulfide, water and primary, secondary and tertiary nitrogen groups. Additionally, a raw count of coordinating O, N, C and S atoms was tracked. The BVS and VECSUM values were calculated using parameters provided in the literature (Brown & Altermatt, 1985 ▶; Brese & O’Keeffe, 1991 ▶; Brown, 2009 ▶). In the case of chloride ions, we did not calculate the BVS and VECSUM values as no published parameters are available.

The formulae for the BVS and VECSUM calculations are
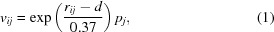


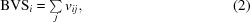


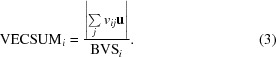
Here, *r_ij_* is the bond-valence parameter for the ion and the co­ordinating atom, *d* is the distance between them, *p_j_* is the percentage occupancy of the ion and **u** is the unit vector pointing from the ion to the coordinating atom. BVS_*i*_ and VECSUM_*i*_ were calculated for each ion identity supported by the SVM where the corresponding *r_ij_* value was available.

When anomalous data were available, we included *f*′′ values that were calculated using *Phaser* (McCoy *et al.*, 2007 ▶; Echols *et al.*, 2014 ▶). Separately, we also trained on the peak height from the anomalous difference map, measured using eight-point interpolation, divided by the expected *f*′′ value to test whether it was possible to avoid the computationally intensive step of determining *f*′′ from the experimental data. For the high-resolution data sets, we omitted the resolution feature owing to the differences in the resolution cutoffs used to create the sets.

#### 2.3.3. Detecting coordination geometry   

To detect coordinating geometries, we used an algorithm to find the shape that best matched the set of coordinating atoms at each site. As an input, this algorithm accepts a list of vertices corresponding to the locations of the coordinating atoms within 2.9 Å around a site. It then begins with a list of possible shapes, each represented by a list of vertices corresponding to that shape. These shapes were first filtered to only include those with an equal number of vertices to the number of coordinating atoms. In both the set of vertices of the shape and the input set of coordinating atoms, the algorithm calculated the angles between each pairwise combination of vertices, using the origin as the middle point. These angles were then sorted in descending order and the r.m.s.d. between the two sorted lists was calculated. The shape with the smallest r.m.s.d. was then selected as the geometry that matches that site. If the r.m.s.d. was above a threshold value for that shape, it also was discarded. The shapes used were taken from common coordination geometries reported in the literature (Harding, 2001 ▶). We have listed the shapes and the parameters included in our algorithm in Supplementary Table S1.

### 2.4. Labeling ions as false positives   

When manually inspecting sites, we followed the previously reported binding and scattering patterns to confirm the identity of ions (Harding *et al.*, 2010 ▶; Echols *et al.*, 2014 ▶). In the case of sodium and chloride ions, the full range of environments has not been fully described anywhere in the literature to the best of our knowledge. For sodium, we added the extra requirement of 5–6 coordinating atoms, a BVS value close to 1 and a VECSUM value below 0.6. As we did not know of any comprehensive rules for chloride coordination, we used the conservative rules that a site have a peak in the *mF*_o_ − *DF*_c_ map and be coordinated by a backbone or side-chain amide group at a distance of around 3 Å from the nitrogen atom.

### 2.5. Filtering SVM predictions   

To apply quality control to the SVM predictions, we added a few simple filters to exclude elements from inclusion in the list of potential sites. Elements were only included as options if (i) the site had a BVS for that element within 50% of any of the charges that may be associated with that element, (ii) the site had a VECSUM below 0.6 for that element, (iii) the site had an *mF*_o_ peak height greater than 1.0, (iv) the site had an *mF*_o_ − *DF*_c_ peak height greater than 0 for elements other than sodium and magnesium, (v) in the case of halides, the site had to be coordinated by at least one positively charged group at a distance of less than 3.5 Å as well as having no negatively charged groups closer than 3.2 Å, and (vi) all nearby atoms were located at least 1.8 Å away from the site. These filters were applied to test sets when mentioned below.

### 2.6. Recall rate   

When used below, the recall rate is defined as the number of correctly labeled sites for an element divided by the total number of sites for that element.

## 3. Results   

### 3.1. Manually curated structures   

#### 3.1.1. Training and test sets   

For the curated structure data set, we obtained 147 structures for SVM training and 138 structures to test its performance (Supplementary Tables S2, S4 and S5). All ions except iron were represented approximately equally in the training and test sets. Iron ions appeared with four times the frequency in the training set as in the test set owing to the limited number of structures meeting the specified criteria.

Within the training set, we observed five examples where a site modeled as water was found to have significant anomalous scattering (defined here as *f*′′ > 1; Supplementary Fig. S2). On closer inspection, we found that two were alternate conformations of a neighboring selenomethionine residue (PDB entries 2i6h and 1pg6). The other three sites (one in PDB entry 2oik and two in PDB entry 2p0n) had three or fewer coordinating atoms and BVS and VECSUM values that did not agree with any of the elements discussed in this paper. These sites may represent inaccuracies in the training data, but because they were not a subset of the elemental ions that were being tested, we chose not to exclude these structures.

#### 3.1.2. SVM classification and feature selection   

In our benchmarks we evaluated the performance of the SVMs in differentiating ions from water molecules as well as pairwise from one another. We found that the SVM trained on curated structures was able to differentiate most ion sites from water, with only nine out of a total of 48 084 water molecules falsely identified as an elemental ion (Table 1 ▶). When we trained an SVM in the absence of anomalous scattering features, the false-positive rate stayed about the same, while the rate of recall dropped slightly. Omitting either entire chemical or scattering environment caused a significant decrease in specificity and a decrease in recall. These data indicate that there are differentiable properties in both types of environments, but that it is important to couple a chemical approach with one that incorporates X-ray scattering. This was especially applicable to calcium ions, that were often bound with partial occupancy resulting in sites that scatter similarly to water.

We next tested how well the SVM could differentiate heavy ions in the curated test set from one another (Table 2 ▶). This method had difficulty in telling the various transition metals apart, as well as manganese from calcium. Omitting anomalous data did not significantly impact the ability of the SVM to differentiate ions from one another. This may be owing to the fact that there were not enough examples of data collected near the X-ray absorption edge for metals in the structure (Supplementary Fig. S2).

#### 3.1.3. Examining the false positives   

To identify what specific types of sites were problematic for the SVM to identify, we examined each site in the test set where the SVM that was trained on all features flagged a water molecule as an ion. Out of seven sites, two are likely correct ion assignments that were overlooked by the original authors; another was attributable to an erroneous initial water placement.

**Figure 2:**
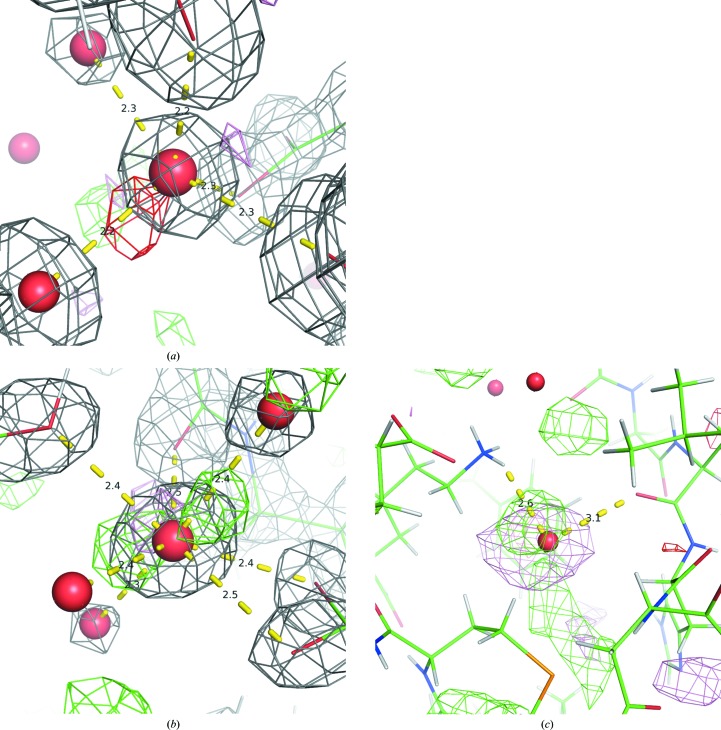
Sites in the curated data set found to be incorrectly modeled as waters. (*a*) PDB entry 2oy2, chain *A*, residue 1290. (*b*) PDB entry 3bwx, chain *A*, residue 629. (*c*) PDB entry 4fca, chain *A*, residue 701. Green and red meshes are *mF*_o_ − *DF*_c_ density at ±3.0σ. The pink mesh is anomalous difference density at 3.0σ. (*a*) and (*b*) include a gray mesh for the 2*mF*_o_ − *DF*_c_ density at 2.0σ. Red spheres are water molecules. Distances are labeled in Å. Images were generated using *PyMOL* v.1.3.

**Figure 3:**
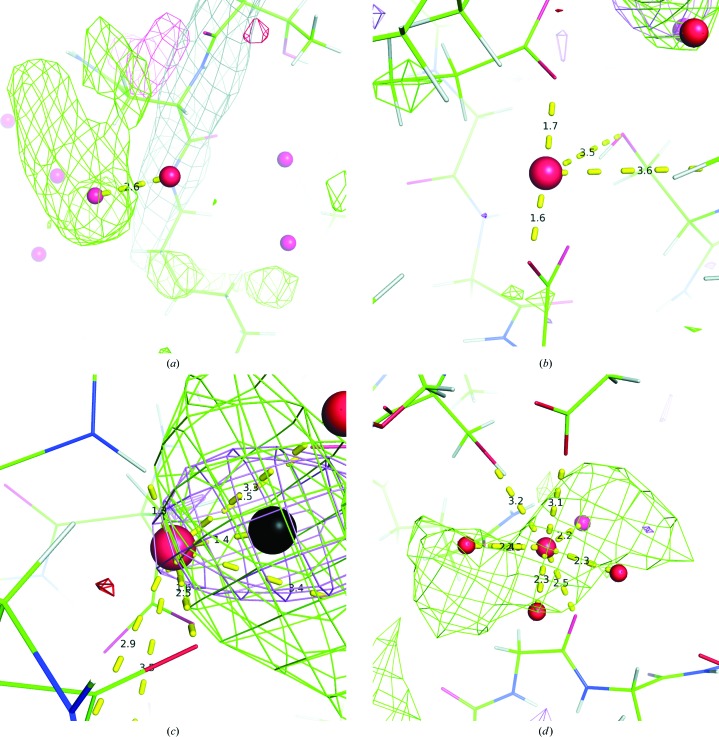
Examples of the different categories of false positives, where a water molecule was incorrectly labeled as a heavy atom by the SVM. (*a*) Site had little or no electron density; PDB entry 2vca, chain *A*, residue 2257. (*b*) Site is coordinated extraordinarily closely by neighboring atoms; PDB entry 3qlq, chain *A*, residue 266. (*c*) Site is coordinating a neighboring heavy atom; PDB entry 3qlq, chain *A*, residue 259. (*d*) Site has an ambiguous environment and could not be successfully identified; PDB entry 2xrm, chain *A*, residue 2024. (*a*) includes a gray mesh for the 2*mF*_o_ − *DF*_c_ density at 2.0σ. Colors, shapes and lines are as in Fig. 3 ▶.

**Figure 4:**
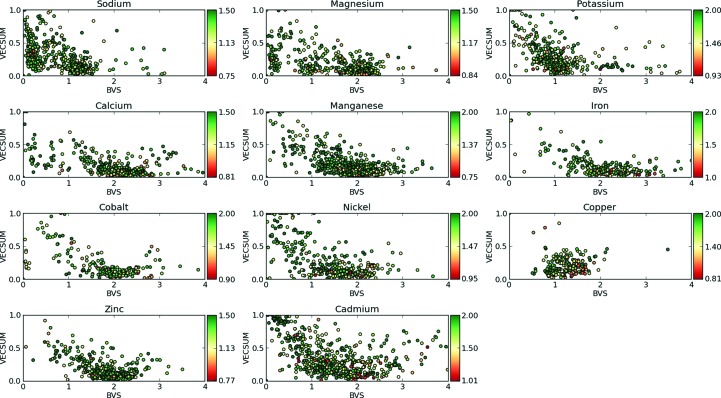
Ion sites have a wide variety of chemical binding environments: BVS values (horizontal axis) plotted against VECSUM values (vertical axis) for ions in each of the high-resolution training sets after re-refinement. Points are colored by structure resolution, with the resolution range for each element indicated by the color bar to the right of the corresponding plot. Outliers with BVS values greater than 4 were omitted for display purposes.

(i) A site in PDB entry 3bwx (Joint Center for Structural Genomics, unpublished work; Fig. 2 ▶ *a*) appeared to be a real calcium site, as its BVS and VECSUM values agreed with its assignment as a calcium. Additionally, it had a distorted pentagonal bipyramid coordination geometry, which has previously been reported to be indicative of calcium sites (Graham *et al.*, 2005 ▶).
(ii) A site in PDB entry 2oy2 (Bertini *et al.*, 2006 ▶; Fig. 2 ▶ *b*) had a trigonal bipyramid coordination geometry and a BVS value that supports the assignment of calcium to the site.
(iii) A site in PDB entry 4fca (Center for Structural Genomics of Infectious Diseases, unpublished work; Fig. 2 ▶ *c*) was identified as nickel but actually appears to be an un­modeled alternate conformation of the neighboring selenomethionine residue.
(iv) A site in PDB entry 2vca (Ficko-Blean *et al.*, 2008 ▶; Fig. 3 ▶ *a*) where a water was identified as a calcium ion although its site had little supporting electron density.
(v) In PDB entry 3qlq (Trastoy *et al.*, 2012 ▶), one site was marked as calcium owing to the unusually close proximity of two carboxyl groups (Fig. 3 ▶ *b*), while another was also marked as calcium owing to a neighboring heavy metal with overlapping density (Fig. 4 ▶ *c*). Both of these sites were predicted to be manganese when calcium was excluded as a possibility.
(vi) In PDB entry 2xrm (Gamble *et al.*, 2011 ▶; Fig. 3 ▶ *d*), a single site with a pentagonal pyramid coordination geometry was identified as a calcium ion. It is possible that this is a correct assignment, as the BVS and VECSUM values for calcium at this site were 2.302 and 0.483, respectively, but the low resolution of the data makes it challenging to confirm this assignment.

In all but PDB entry 3xrm, the use of conservative cutoffs, such as a minimum *mF*_o_ peak height or a maximum BVS value, would have eliminated the incorrect ion assignments. We examine this use of *a priori* information later in this work.

### 3.2. Automatically curated high-resolution structures   

#### 3.2.1. Training and test sets   

For each ion, the high-resolution training sets contained between 87 and 330 structures. The total ion counts in both the training and test sets ranged from 218 for cobalt ions up to 825 for chloride ions (Supplementary Tables S3 and S6). All ions had approximately two to three times as many sites within the training set as the test set. Consistent with the findings of Müller *et al.* (2003 ▶), we observed a wide distribution of BVS and VECSUM values for all ions in these structures, despite the relatively high resolutions (Fig. 4 ▶). In most cases the distribution is densest around the expected bond valence (one for Na and K; two for Mg and most heavier ions), but the clustering of outliers suggests that a large fraction of input ions may either be mis-assigned or have malformed binding sites (for example, missing coordinating waters). The practical implications for our method are discussed below. We also collected simple statistics on the frequencies of coordination environments (Supplementary Tables S10 and S11) and found that the ion-binding patterns in the training set were similar to those previously reported in the literature (Zheng *et al.*, 2008 ▶; Harding *et al.*, 2010 ▶).

#### 3.2.2. SVM classification   

Within each high-resolution data set, we tested how well the SVMs trained on unexamined high-resolution structures could differentiate their respective ion from structured water molecules. For most ions, the relevant SVM had a recall rate above 65% and a low false-positive rate (Table 3 ▶). Recall rates were lower for sodium, chloride and potassium ions, which is most likely owing in part to in­accuracies in deposited structures or possibly the fact that the binding sites and scattering properties tend to be more similar to water. Upon visual inspection, we found that many of the original water sites unexpectedly flagged as sodium and chloride ions by the SVM were in fact genuine examples of those respective elements binding (Supplementary Table S12); this is consistent with previous reports that sodium and chloride ions are commonly mislabeled (Dauter & Dauter, 2001 ▶).

We also combined the list of high-resolution ion sites to test how well a SVM could differentiate ions from one another (Supplementary Table S9). Overall, the results were positive: out of the 66 pairwise combinations of ions tested, 53 had a precision above 80% for both ions and 38 had a precision above 90%. On average, the SVM had a precision of 91% and a recall of 65%, compared with 89 and 34%, respectively, for our previously reported method (Echols *et al.*, 2014 ▶). There were three cases where the accuracy of the SVM was consistently lower than the average. (i) As expected, transition metals were consistently difficult to tell apart. (ii) Magnesium and calcium ions were frequently mislabeled as sodium ions. This may be owing to the fact that all three bind similar environments and scatter with similar intensities. However, the spread of valences for these two ions are quite wide (Fig. 4 ▶) and it is possible that this is attributable to mislabeled sites in the test sets. (iii) Iron ions had an increased chance of being labeled as sodium. We do not have an explanation for this last case and can only note that it did not appear between iron and other nontransition metals. Thus, SVMs are capable of telling ions apart from both water as well as most other ions, even when no manual curation was applied to their training data.

#### 3.2.3. Examining the false positives   

As above, for each site in the test set that was originally modeled as a water molecule but flagged as an ion by an SVM, we manually inspected its environment to confirm that it was indeed a false positive (Supplementary Table S12). We found that out of 208 sites flagged in this manner by an SVM, 96 were true examples of unmodeled ions (Table 3 ▶). Out of these 96 sites, 22 were unmodeled ions whose true identities were not what the SVM was originally trained on. For the 112 remaining false positives we noticed many of the same trends as in the curated test set: 16 sites were labeled as an ion when they had little to no supporting electron density and 36 sites were coordinated too closely (<2.0 Å) by the neighboring atoms to support any ion assignment. Additionally, 28 sites coordinated another ion of the same charge, but this was not detected since all ions in the test set were relabeled as water molecules before refinement.

### 3.3. SVM classification compared with a decision tree   

To compare the trained SVMs against our previous method, we ran the decision-tree algorithm from our previous paper (Echols *et al.*, 2014 ▶) on each high-resolution test set as well as the manually curated test set (Tables 1 ▶, 2 ▶ and 3 ▶ and Supplementary Table S9). Across every data set, SVMs out­performed the decision tree, often finding twice the number of model ions. However, this increased sensitivity came at the cost of reduced specificity. In the high-resolution test set SVMs flagged 112 water molecules as ions, three times more than the 31 water molecules flagged as ions by the decision-tree method.

The decision-tree method was primarily optimized on structures containing calcium and zinc. However, it was also designed to be relatively permissive towards heavy ions with respect to geometry when anomalous scattering and/or difference map peaks were present. In line with the results using the manually curated set (§[Sec sec3.1.2]3.1.2), the omission of anomalous scattering information in the high-resolution automatically curated test sets did not limit the performance of these SVMs. This confirms that there is redundant information for classification when not specifically comparing between different ions.

#### 3.3.1. A combined approach   

Based on our observations of the patterns in the false positives flagged by SVMs, we tested the effect of adding a few basic filters on SVM predictions (see §[Sec sec2]2). By applying this combination to the high-resolution test set, we were able to eliminate 82% of the false positives while reducing our recall rate by 13% (Table 3 ▶). Additionally, when applied to comparisons between ions, the number of ions falsely predicted to be another ion decreased by 16%, although the recall decreased by 12% as well, resulting in almost no change in average precision (Supplementary Table S9).

Up to this point, although we discarded entire structures based on global refinement statistics, we did not apply any filtering to the individual sites in the high-resolution training set. However, our simple assessments of the training set showed many sites with implausible scattering and/or chemical environments (Fig. 4 ▶; Supplementary Fig. S3). In order to assess the quality of these sites, we applied similar filters for quality control on the training inputs, using information from valences and electron density as in the outputs above. After applying these filters to the high-resolution training set, we observed a significant change in the ion count for sodium, magnesium and chloride, with drops of over 100 for each, but little change for most other ions (Supplementary Table S3). When differentiating ions from water, we found that this approach reduced the total number of false positives by 19% and the recall by only 4% (Table 3 ▶). Combined with filters on the SVM outputs, we saw a minor improvement: 17 false positives, down from 20, and a drop in the recall of only 3%.

To assess the confidence of the probabilities assigned by the SVM to its predictions, we calculated the ratio of the score of the predicted ion divided by the sum of the scores for all other ions when evaluating all possible ions at a site (Supplementary Fig. S5). We found that although the ratios for incorrect assignments tended to fall below 1.0, the distribution was skewed to the right, with many incorrect assignments having scores at least five times greater than that of all other ions combined. Although this suggests that there is some value to screening by ‘confidence’, it did not prove to be as effective as the other filters discussed above.

Overall, these results show that simple filters based on *a priori* knowledge of chemical and scattering patterns of ions are essential to maintaining a low false-positive rate when differentiating ion sites from structured water molecules. However, they are still not adequate to differentiate all ions from one another with the same degree of accuracy: more experimental data, and intelligent algorithms that act upon anomalous information, are required for this task (Mueller-Dieckmann *et al.*, 2007 ▶; Thorn & Sheldrick, 2011 ▶; Echols *et al.*, 2014 ▶).

## 4. Discussion   

Pattern-recognition techniques applied to electron-density maps have previously been shown to be a powerful tool for sequence assignment in automated protein model building (Gopal *et al.*, 2007 ▶; Langer *et al.*, 2008 ▶). They have also proven useful for identifying possible metal-binding sites from structure alone (Bordner, 2008 ▶; Buturovic *et al.*, 2014 ▶). Here, we trained SVMs on information from the X-ray scattering and local chemical environment. These SVMs were able to reliably predict the identity of the 12 most common ions in the PDB at moderate to high resolutions, with the exception of sodium. In the case of sodium, the inability of our method to select many useful features for classification (Supplementary Table S8) and the wide spread of valences (Fig. 4 ▶) suggest that this ion is frequently misclassified. This highlights the need for additional curation and correction of ions in existing structures before machine-learning methods may be used.

The method described in this paper also provides a straightforward route to improve its predictive ability as more metal-bound structures are deposited. Although the trained SVMs were unable to reliably tell transition-metals ions apart without the use of anomalous scattering near elemental *K* edges, they were able to differentiate calcium ions from zinc, nickel and manganese with a high degree of accuracy in the curated test set (Table 2 ▶). Additionally, within the high-resolution test sets, an SVM was able to differentiate most chemically distinct ions with an average precision and recall of 91 and 65%, respectively, improved from precision and recall rates of 89 and 34% when using the method of decision trees (Supplementary Table S9).

### 4.1. Comparison of SVMs and manual decision trees   

Although the manually constructed decision tree was shown to be applicable to ion identification in our previous work, it is inherently limited by the ability of the designer to set optimal parameters and to pick out relationships between multiple features simultaneously. For each additional metal, new parameters must be determined and later updated as more structures containing that metal are published. These parameters are often based around *a priori* knowledge that leads to sensible results, such as coordination chemistry and anomalous scattering that are consistent with those reported in the literature. However, the allowable range for these features is set at the discretion of the methods developer. Even structures with a resolution of 2.0 Å or better were found to have ion BVS values that deviated by greater than 50% from their ideal values (Fig. 4 ▶; Müller *et al.*, 2003 ▶). Ion electron-density and difference map peak heights were also found to deviate by up to 200% from their average values (Supplementary Fig. S3).

This process of optimizing cutoffs is time-consuming, and often the associations between multiple features are not apparent from a superficial inspection. This leads to an algorithm that is both too permissive in some cases and too stringent in others, and excellent coordination chemistry for one ion is not used as information to rule out other ions. For instance, multiple structures have been identified in which calcium is coordinated by nitrogen, an event considered to be an outlier in previous reports in the literature (Zheng *et al.*, 2008 ▶). Tuning the cutoffs to catch these corner cases while maintaining a low false-positive rate requires many iterations of cross-validation.

To solve this problem of tuning parameters, we turned to SVMs to automatically determine the best cutoffs for each feature and how different features may be used in conjunction with one another. Although they have shown to be successful for our purposes, they are not without drawbacks. While their strength lies in their ability to quickly and automatically identify predictive features for classification problems, they are nonetheless limited by metaphorically driving *via* the rear-view mirror: because they rely on a pre-selected set of example ion sites, they may be inaccurate when predicting ions in novel or uncommon environments. While we have attempted to control for this overfitting by removing non­predictive features, the potential still remains for inaccurate assignments. We saw three examples of this in PDB entry 2vca (Fig. 3 ▶ *a*), where the water molecules placed in low electron density were incorrectly identified as calcium ions. Additionally, SVMs do not solve the problem of disambiguating the transition metals, which appear to be similar to one another in both their chemical and scattering environments. Indeed, when manually fitting these metals it is expected that a crystallographer collect anomalous data near the X-ray absorption *K* edges of the metals (Read & McCoy, 2011 ▶; Echols *et al.*, 2014 ▶; Zheng *et al.*, 2014 ▶) and/or employ complementary techniques for element identification (see, for example, Garman & Grime, 2005 ▶; Shi *et al.*, 2005 ▶; Bergmann & Glatzel, 2009 ▶).

Ultimately, SVMs are limited by the quantity and quality of the training input. A number of elements are less pervasive in the PDB (Supplementary Fig. S1), and finding enough high-quality structures to train a SVM to reliably differentiate them from both water and other ions can be challenging. In the case of sodium ions in particular, there are many spurious examples which impeded the ability of the algorithm to find patterns in the data. Automated curation of these data sets is effective in reducing the false-positive rate, but risks reducing sensitivity as well, especially at lower resolutions where the coordination geometry is often imperfect or incomplete. We note, however, that in any tests against automatically curated data the true ‘false-negative’ rate is likely to be significantly lower than that presented here owing to the presence of spurious ions in the test sets.

For optimal classification, we have found that it is most efficient to combine these two approaches by using SVMs to mine the predictive features of a site and a more simplified decision tree as a mechanism of quality control. Simple rules can prevent the SVM from suggesting ions that would immediately be rejected by a crystallographer. These rules include rejecting sodium and magnesium from sites with anomalous signal, rejecting ions that give large VECSUM values and requiring that sites have significantly larger *mF*_o_ signal than other water molecules for the placement of heavy atoms. Additionally, the classification described here is applied independently to each ion and does not automatically consider the scenario where two adjacent atoms are flagged as possible ions with like charges (for example when multiple water molecules are placed in density for a heavier atom). In the context of our automated refinement procedure (Echols *et al.*, 2014 ▶), this is easily corrected by sorting all candidate ions by descending 2*mF*_o_ − *DF*_c_ map level and ignoring any atoms adjacent to an already placed ion.

### 4.2. Future directions   

One question that remains largely unexplored is whether SVMs can be used to classify rarer metals and halides. Although our method has some applicability to distinguishing chloride ions from water and various cations, halide ions are particularly difficult to identify owing to their nonspecific binding patterns, which may include hydrophobic contacts (Dauter & Dauter, 2001 ▶). Our method may be extended to these ions if enough correctly labeled examples are present, but we have not tested its accuracy on this problem here. We also have not explored the use of more complex features, such as the electrostatic potential and solvent-accessible surface area at a site, the shape of the electron-density peak or the local peptide sequence, which have been shown to be associated with metal-binding sites (Brylinski & Skolnick, 2011 ▶; Carugo, 2008 ▶, 2014 ▶). While relatively computationally intensive, these features may improve the predictive power of the classifier.

Not surprisingly, one of the major limitations of our approach is the unreliability of many structures in the PDB even at high resolution, and continuous and large-scale manual curation of these structures by experts is prohibitively time-consuming. Although improvements to validation methods are helpful (see, for example, Zheng *et al.*, 2014 ▶), it is difficult to formulate rules that will apply across a wide resolution range without excluding a large number of valid sites, and any method for comprehensively judging crystal structures must take experimental data into account. Continuous re-evaluation of structures, especially in the context of improved computational methods (Joosten *et al.*, 2012 ▶), may help with the curation of large data sets of published structures, but the deposited data are rarely sufficient to unambiguously identify many elements. In any case, any decision about the identity of a site should currently not be made by software alone, and must incorporate external information beyond the inputs used here. Although computational methods are effective at reducing manual effort in the building and refinement workflow, a comprehensive and scientifically robust approach requires the integration of multiwavelength data sets and/or complementary experiments into the data-collection, processing and deposition pipelines.

## 5. Availability   

The trained SVMs and accompanying programs are available as part of *PHENIX* v.1.9.1 or more recent, which is free of charge for academic users (http://phenix-online.org/). Source code is included in the distribution; additional code and the data sets used for training are available from the authors.

## Supplementary Material

Supporting Information including Supplementary Figures S1-S5, Supplementary Tables S2 and S3, and captions for all other Supplementary Tables.. DOI: 10.1107/S1399004715004241/tz5065sup2.pdf

Supplementary Table S1.. DOI: 10.1107/S1399004715004241/tz5065sup1.xlsx

Supplementary Table S4.. DOI: 10.1107/S1399004715004241/tz5065sup3.xlsx

Supplementary Table S5.. DOI: 10.1107/S1399004715004241/tz5065sup4.xlsx

Supplementary Table S6.. DOI: 10.1107/S1399004715004241/tz5065sup5.xlsx

Supplementary Table S7.. DOI: 10.1107/S1399004715004241/tz5065sup6.xlsx

Supplementary Table S8.. DOI: 10.1107/S1399004715004241/tz5065sup7.xlsx

Supplementary Table S9.. DOI: 10.1107/S1399004715004241/tz5065sup8.xlsx

Supplementary Table S10.. DOI: 10.1107/S1399004715004241/tz5065sup9.xlsx

Supplementary Table S11.. DOI: 10.1107/S1399004715004241/tz5065sup10.xlsx

Supplementary Table S12.. DOI: 10.1107/S1399004715004241/tz5065sup11.xlsx

## Figures and Tables

**Table 1:** Benchmark of the ability of classifiers to differentiate each ion from water in the blind, curated test set Shown here are the unmodified decision-tree algorithm as well as SVMs trained on the curated training set using (i) all features of a site, (ii) all features except anomalous scattering, (iii) only electron density and anomalous scattering information and (iv) only information about the chemistry of a site. The pairs of numbers for each SVM and ion indicate the number of true positives (ions correctly identified) and false positives (water molecules identified as ions).

Ion *versus* water	Structures	Ions	Waters	Decision tree	All features	Anomalous peak	No anomalous	Scattering only	Chemistry only
Ca^2+^	53	108	18384	60, 0	92, 6	84, 7	89, 6	91, 24	63, 7
Mn^2+^	33	70	9632	31, 1	68, 2	65, 1	64. 2	67, 0	62, 4
Fe^2+/3+^	14	17	3077	9, 0	17, 0	16, 0	16, 1	17, 0	15, 1
Ni^2+^	26	49	11923	32, 1	48, 1	47, 0	48, 0	49, 3	42, 7
Zn^2+^	24	64	12651	47, 0	60, 0	56, 0	56, 0	59, 0	55, 0
Total true positives				179	285	268	273	283	237
Total false positives				2	9	8	9	27	19

**Table 2:** Benchmark of the abilities of classifiers to distinguish different ions from one another in the blind, curated test set Shown here are the unmodified decision-tree algorithm as well as SVMs trained on the curated training set using all features of a site and the same SVM with a simple filter applied to the predictions. The pairs of numbers for each SVM and ion pair indicate the number of correct identifications of the first and second ion, respectively.

		Total sites		Decision tree		All features		All features (filtered predictions)	
Ion 1	Ion 2	Ion 1	Ion 2	Ion 1	Ion 2	Ion 1	Ion 2	Ion 1	Ion 2
Ca^2+^	Mn^2+^	108	70	60, 0	31, 2	86, 9	59, 8	84, 5	61, 10
Ca^2+^	Fe^2+/3+^	108	17	60, 0	9, 0	92, 0	17, 0	90, 0	14, 1
Ca^2+^	Ni^2+^	108	49	60, 0	32, 0	91, 0	48, 1	90, 0	38, 2
Ca^2+^	Zn^2+^	108	64	60, 0	47, 0	92, 0	60, 0	90, 0	52, 1
Mn^2+^	Fe^2+/3+^	70	17	11, 1	6, 0	51, 2	15, 17	50, 2	12, 16
Mn^2+^	Ni^2+^	70	49	22, 0	28, 0	52, 2	46, 16	51, 7	36, 15
Mn^2+^	Zn^2+^	70	64	26, 0	33, 0	46, 3	57, 22	45, 5	50, 21
Fe^2+/3+^	Ni^2+^	17	49	2, 0	27, 0	11, 5	43, 6	8, 9	33, 6
Fe^2+/3+^	Zn^2+^	17	64	2, 0	35, 1	11, 2	58, 6	9, 3	50, 5
Ni^2+^	Zn^2+^	49	64	1, 0	24, 0	18, 5	55, 30	17, 3	49, 21
Total true positives				569		1008		929	
Total false positives				4		134		132	

**Table 3:** Benchmark of the ability of classifiers to differentiate each ion from water in the automatically curated test set Shown here is the unmodified decision-tree algorithm as well as SVMs trained on all features except anomalous scattering and resolution. We have included statistics for SVM classification as well as classification when stringent inputs were used for SVM training and classification when a simple filter was applied to the list of elements used in prediction. Numbering follows the same pattern as in Table 1 ▶.

Ion *versus* water	Structures	Ions	Waters	Decision tree	SVM	SVM (filtered predictions)	SVM (stringent inputs)	SVM (stringent inputs, filtered predictions)
Na^+^	95	196	43498	32, 9 (28 Na^+^)	52, 7 (42 Na^+^)	46, 0 (41 Na^+^)	8, 5	6, 0
Mg^2+^	87	206	49603	111, 1 (7 Na^+^, 2 Mg^2+^, 1 Ca^2+^)	144, 10 (3 Mg^2+^, 2 Cl)	128, 4 (3 Mg^2+^)	127, 4 (5 Mg^2+^)	125, 2 (5 Mg^2+^)
Cl	192	492	103229	77, 3 (21 Cl, 2 I)	260, 15 (22 Cl, 1 K^+^, 2 I)	199, 9 (17 Cl, 1 K^+^, 2 I)	257, 17 (24 Cl, 2 K^+^, 2 I)	195, 10 (21 Cl, 2 I)
K^+^	96	259	48569	73, 3 (4 K^+^)	148, 11 (1 Cl, 4 K^+^, 2 I)	118, 2 (3 K^+^)	145, 8 (2 Na^+^, 3 K^+^, 2 I)	116, 2 (3 K^+^)
Ca^2+^	93	244	46547	64, 0 (2 Ca^2+^)	188, 11 (1 Cl, 2 Ca^2+^)	172, 3 (2 Ca^2+^)	189, 7 (2 Ca^2+^)	172, 3 (2 Ca^2+^)
Mn^2+^	95	284	49859	158, 4	235, 25	223, 2	232, 23	223, 0
Fe^2+/3+^	93	320	46960	154, 1	199, 2	180, 0 (1 Cl)	203, 1	182, 0
Co^2+^	47	135	27716	38, 0	89, 13 (1 Mg^2+^)	77, 0 (1 Mg^2+^)	86, 6	75, 0
Ni^2+^	48	93	21531	30, 2	68, 2	48, 0	66, 4 (1 Ni^2+/3+^)	48, 0
Cu^+/2+^	41	92	16076	30, 0	74, 3	73, 0	75, 3	74, 0
Zn^2+^	95	289	41939	173, 0	264, 7 (2 Cl, 1 Zn^2+^)	243, 0	265, 6 (2 Cl)	245, 0
Cd^2+^	45	234	15981	132, 7 (1 Cd^2+^)	178, 6 (7 Mg^2+^, 3Cd^2+^)	143, 0 (1 Cd^2+^)	178, 7 (6 Mg^2+^, 1Cd^2+^)	141, 0 (1 Cd^2+^)
Total true positives				1072	1899	1650	1831	1602
Total false positives				31	112	20	91	17
Discovered ions				73	96	72	52	34
