# Supplementary material for: Using support vector machines to improve elemental ion identification in macromolecular crystal structures
Source: Acta Crystallogr D Biol Crystallogr. 2015 Apr 25;71(Pt 5):1147–58. doi: 10.1107/S1399004715004241 (PMC4427199; doi:10.1107/S1399004715004241)

BIOLOGICAL
CRYSTALLOGRAPHY

Volume 71 (2015)

Supporting information for article:

Using support vector machines to improve elemental ion identification in macromolecular crystal structures

Nader Morshed, Nathaniel Echols and Paul D. Adams

Table S1 List of geometries recognized by the geometry labeling algorithm.

Included are the parameters for the number of atoms for each geometry group, the RMSD threshold above which geometry labels are not matched to an experimental coordination geometry, and a description of the shape in cases of unconventional geometries. Bidentate groups replaced the single vertex by two vertices separated by an angle of 130°.

Table S2 Training set statistics for curated data set where anomalous data was required, broken down by ion identity.

Structures column counts the number of structures in the training set that contain at least one example of that ion. Sites column counts the total number of example ion and water sites in the data set.

Ion	Structures	Sites
Water	147	48084
Ca^{2+}	51	126
Mn^{2+}	31	67
Zn^{2+}	41	95
$Fe^{2+ / 3+}$	22	64
Ni^{2+}	23	70

Table S3 Training set statistics for uncurated, high resolution data set, broken down by ion identity.

The structures column indicates the number of models in the training set that contain at least one example of that ion. Waters and Ions columns count the total number of sites containing that molecule in each data set.

Ion	Structures	Waters	Ions	Ions (Stringent)
Na^{+}	232	100000	486	158
Mg^{2+}	247	140507	563	241
Cl^{-}	330	150000	825	797
K^{+}	182	106909	450	432
Ca^{2+}	256	133094	677	658
Mn^{2+}	256	133927	688	662
$Fe^{2+ / 3+}$	162	82341	267	267
Co^{2+}	87	35167	217	203
Ni^{2+}	170	69101	366	362
**Cu^{2+}**	114	50942	220	219
**Zn^{2+}**	207	91889	530	522
**Cd^{2+}**	103	39124	585	581

Table S4 Comments for each structure that was either rejected or accepted into the manually curated data set.

Note that this only includes structures that were successfully re-refined and passed the general model quality cutoffs.

Table S5 Full list of PDB IDs used in the curated training and test sets, includes information on resolution, and counts of each ion and water.

Note that this list does not include structures that did not make it to the step of SVM training.

Table S6 Full list of PDB IDs used in the uncurated, high resolution training and test sets, includes information on resolution, and counts of each ion and water.

Note that this list does not include structures that did not make it to the step of SVM training.

Table S7 Rankings of each feature for each SVM applied to the curated training set, using recursive feature elimination (Guyon et al., 2002).

Larger numbers indicate a lower importance in predicting ion identity. In cells where a number is not listed, there appeared no variation in that feature across that entire data set.

Table S8 Ranking of each feature for each SVM applied to the high resolution training set, using recursive feature elimination (Guyon et al., 2002).

Larger numbers indicate a lower importance in predicting ion identity. In cells where a number is not listed, there appeared no variation in that feature across that entire data set.

Table S9 Benchmark of SVMs' ability to differentiate pairs of ion classes within the high resolution, uncurated test set.

Included are the number examples of each class, the number of sites correctly identified for each class, and the associated precision and recall rates. See Table 3's caption for more notes.

Table S10 Frequencies of coordination of ions by each unique chemical group in the automatically curated, high resolution training set.

Percentages here indicate the number of sites where an ion is coordinating by at least one chemical group of a given type divided by the total number of sites for that ion.

Table S11 Frequency of coordination geometries in the automatically curated, high resolution training set.

Percentages here indicate the number of sites that geometry was the bit fit for the coordinating atoms at a site. We have omitted all geometries that had frequencies under 10% for all ions.

Table S12 PDB IDs, site labels, and notes for each water site labeled as an ion by a SVM or by the previous method.

Rows that are italicized were still predicted to be that ion after the filters on the SVM outputs.

Figure S1 Number of modeled ions in the PDB, as of September 22nd, 2014 (based on Echols et al. 2014). This does not include instances of these elements as part of other molecules (e.g. heme, chlorophyll, or iron–sulfur clusters). Only ions that are present in at least 300 structures are shown here. All oxidation states of each metal are counted. Pink bars represent the counts of all deposited structures containing the specified ions; green bars are for structures filtered at 90% sequence identity.

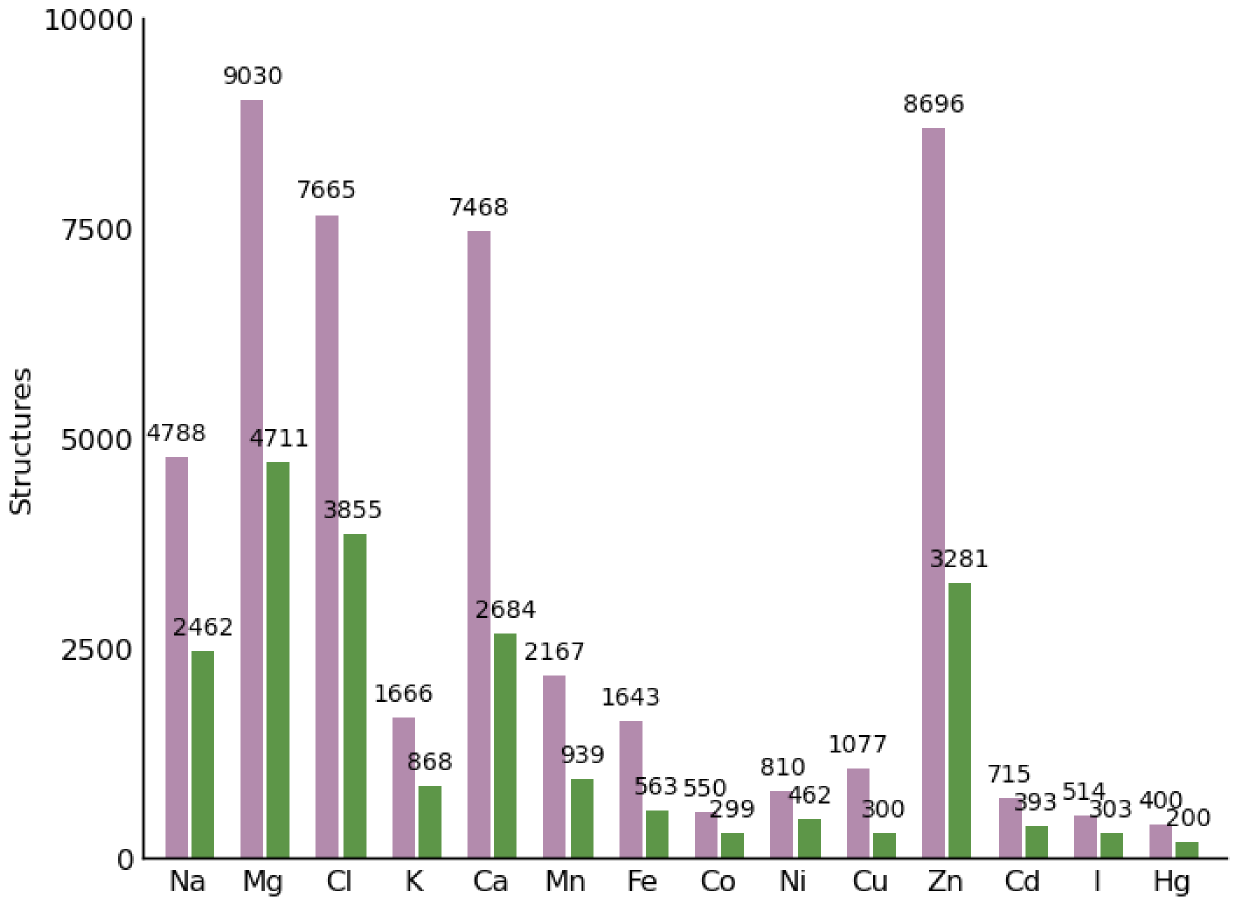

Figure S2 Distribution of f’’ versus wavelength in the curated training set. Colors are orange for manganese, black for zinc, green for iron, red for nickel, grey for calcium, and blue for water. Also indicated are the expected f’’ values for metals with significant anomalous scattering in this wavelength range (Solid lines).

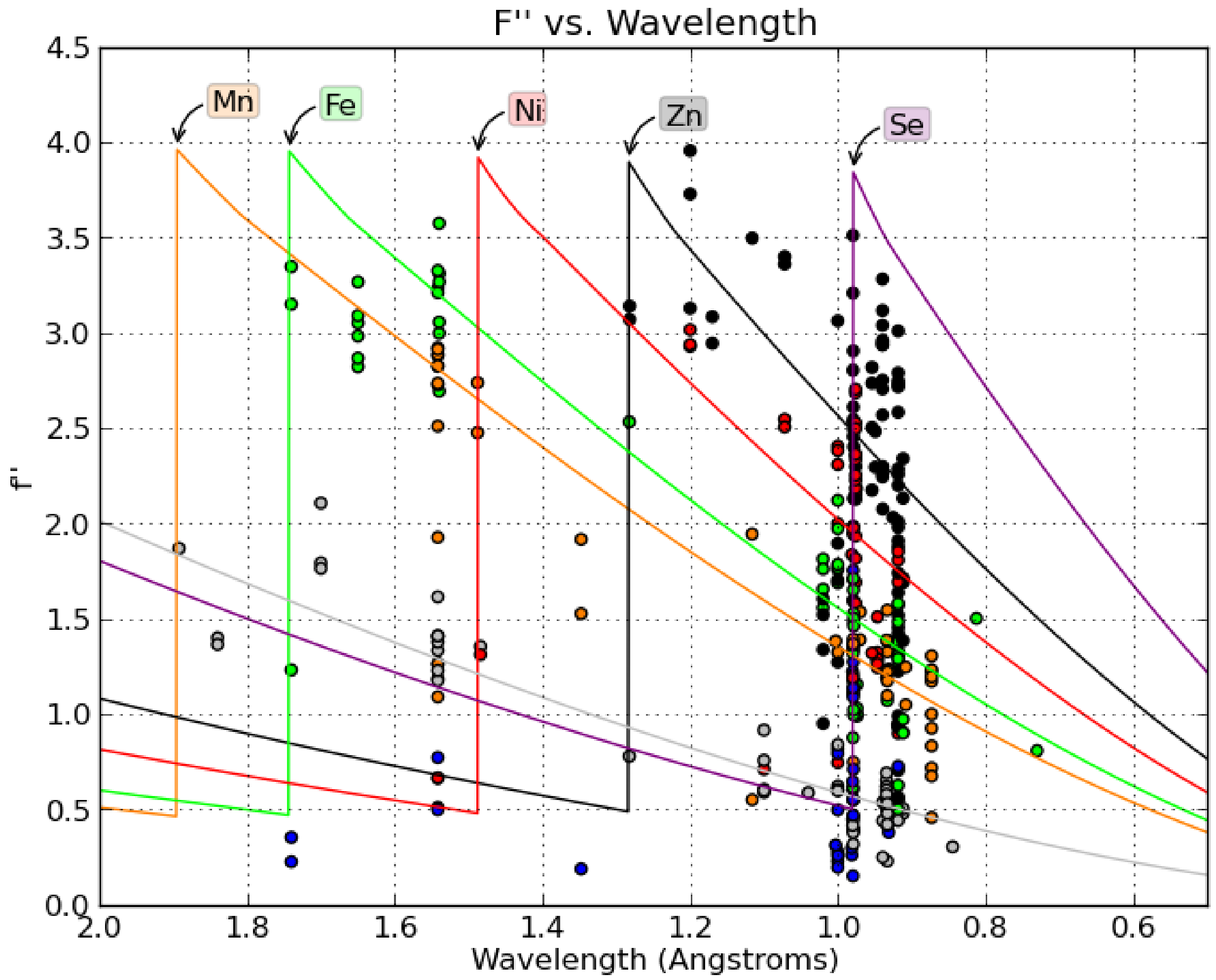

Figure S3 Histogram of electron density peak heights for ions in the high resolution training set. Shown here are the heights a Gaussian function fit to the *mF* map (blue) as well as the exact peak height of the mF_o-DF_c map at the position of the ion in the unit cell (green).

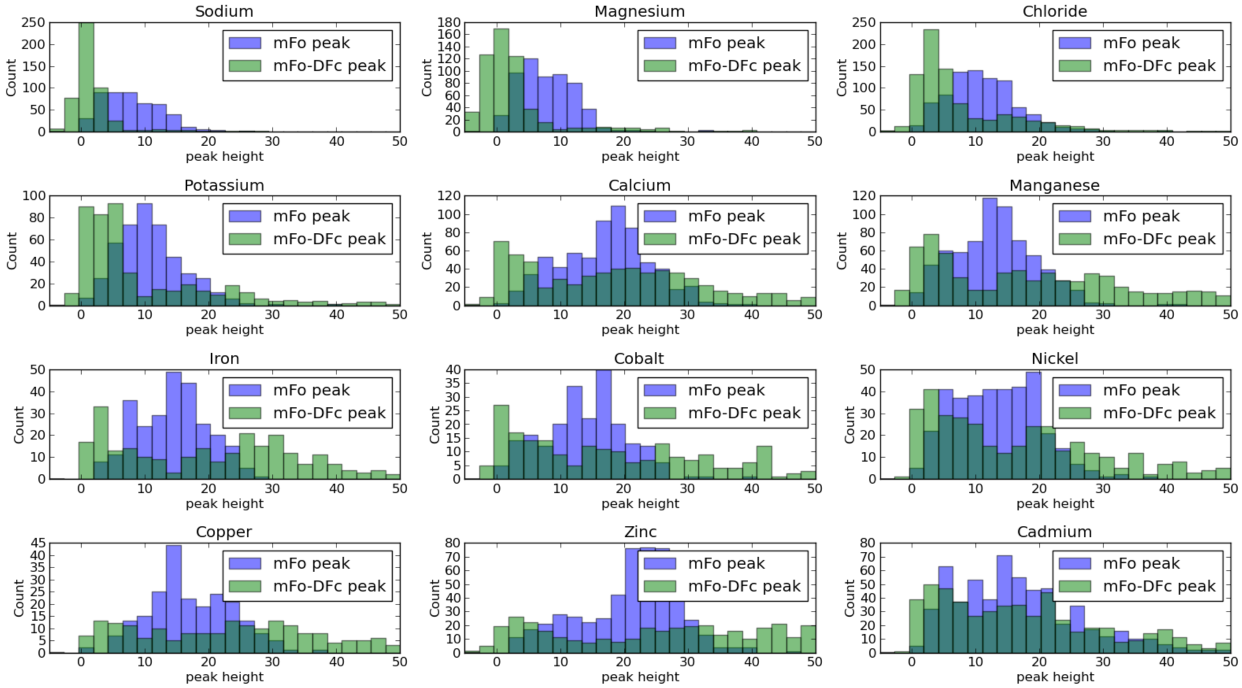

Figure S4 ROC curve showing the true positive rate versus the false positive rate when using each SVM, trained on high resolution structures, to differentiate ions from water in the relevant ion's test set.

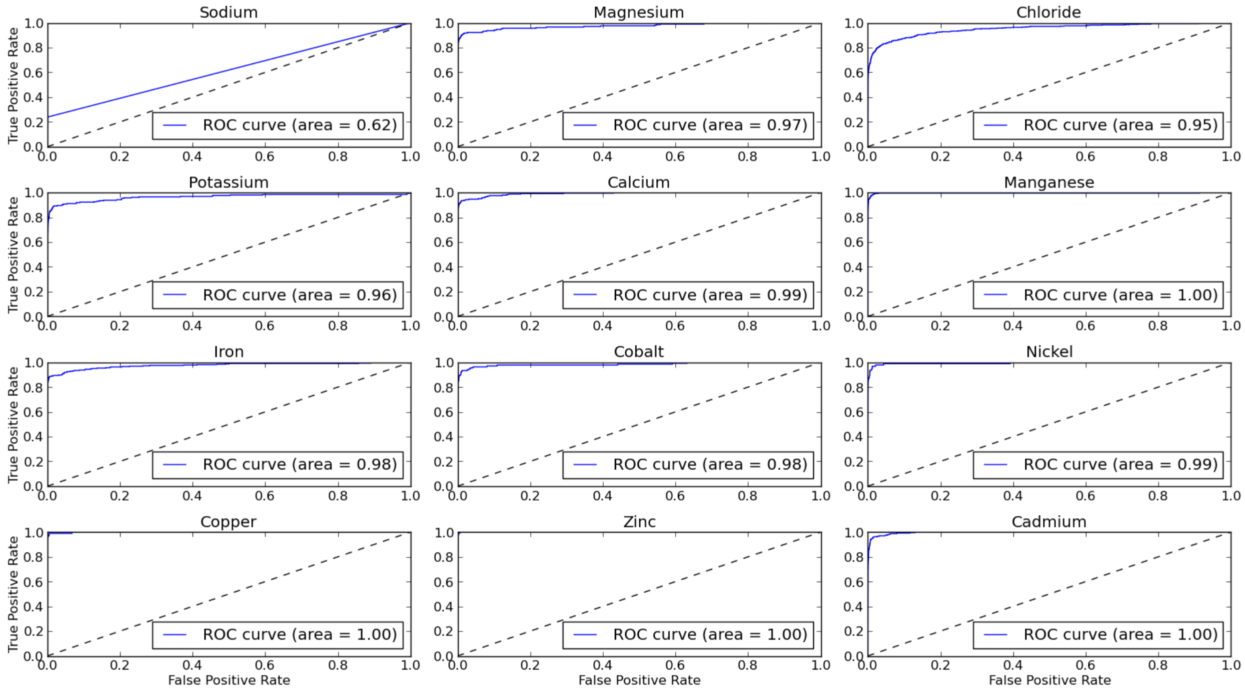

Figure S5 Histogram of the ratios between the SVM score for the predicted ion and the scores for all other ions for both correct (green) and incorrect (red) SVM predictions. These ratios only include the cases where a site was marked as the specific ion labeled for each box. Outliers with ratios greater than 5 were omitted for display purposes.

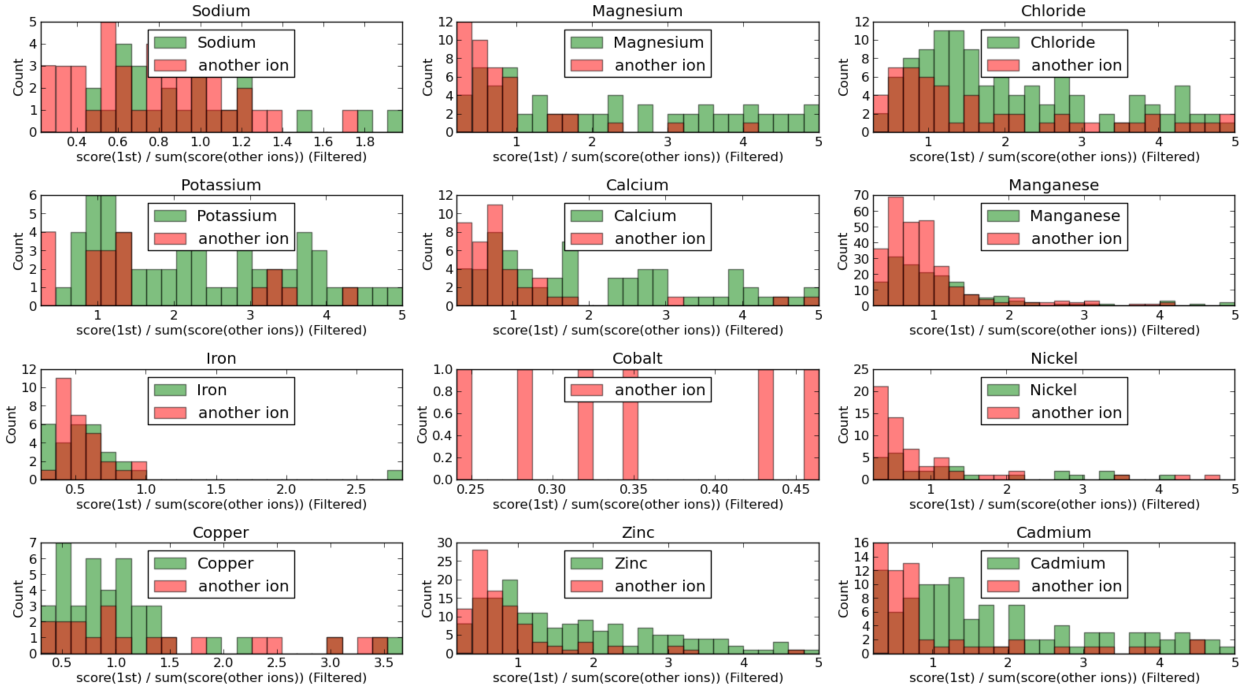

Supplement: Supplementary file 1 [file d-71-01147-sup2.pdf]
